# Are We Underestimating Species Extinction Risk?

**DOI:** 10.1371/journal.pbio.0030253

**Published:** 2005-06-07

Aside from global climate change, loss of biodiversity poses one of the greatest threats to the planet. Last year, the World Conservation Union reported an unprecedented decline in biodiversity, with nearly 16,000 species facing extinction. The biggest threat to the vast majority of these species is loss of habitat. And as habitat loss and degradation proceed nearly unabated, the need to accurately predict the population dynamics and extinction risk of potentially endangered species has never been greater. In a new study, John Drake tests models traditionally used to estimate the likelihood of extinction and shows that because the models ignore a critical parameter in projecting risk, they underestimate extinction rates.

Standard models for predicting extinction assume that population growth and decline are governed by random, or stochastic, variables. The models typically incorporate two major contributors to random variation in population growth rates: changes in environmental conditions and chance fluctuations in population size—caused by variations in individual fitness, random mating behavior, and events that affect just one individual—that are referred to as demographic stochasticity. But since few scientists have tested these models with empirical data, the question remained whether the models were accurately predicting population fluctuations and extinction risk.

To test the reliability of standard stochastic models, Drake used data from experiments with water fleas. He found that the models could accurately predict extinction risk only when there was enough information about variation in individual fitness to account for demographic variability—a finding that undercuts the conventional wisdom that demographic stochasticity is unimportant. Some traditional models do not even include demographic stochasticity.

It's generally assumed that fluctuating environments, a given in the natural world, increase a species's chance of extinction. Drake tested this notion in experiments by manipulating the available food sources in 281 populations of water fleas. The flea populations received either low, medium, or high amounts of food, and Drake kept daily tallies of population number and extinctions. When he tried to predict the extinctions using traditional models, he couldn't. [Fig pbio-0030253-g001]

**Figure pbio-0030253-g001:**
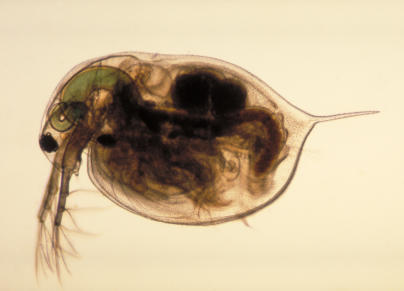
Experiments with Daphnia magna, the water flea, show that traditional extinction models may be underestimating extinction risk

To account for the discrepancy between model and data, Drake began to investigate a possibility raised by recent theoretical research that population density and individual interdependence might affect a major component of the model—demographic stochasticity. The idea is that if organisms interact in their environments—which of course they do—then these interactions will likely affect an individual's probability of dying or reproducing, which ultimately affects species survival. Drake calls this variable density-dependent demographic stochasticity.

Drake used half of the experimental data generated from testing the effects of environmental variability on water flea survival to select his models and estimate the range of parameters that might affect extinction, and the other half to test the models' reliability. From the estimated parameters, Drake wrote a computer program to simulate all the possible population outcomes and predict extinction rates. One set of simulations included a parameter for density-dependent random interactions and another did not.

When Drake analyzed all the possible outcomes, it turned out that manipulating food supply didn't have as great an effect on extinctions as predicted—possibly because individual water fleas live too long compared to the frequency of the environmental fluctuations. Only when density-dependence was included did the models match the observed extinction rates in the flea experiments. When density dependence was not included, extinction rates were greatly underestimated.

Drake's results underscore the importance of bolstering extinction models with empirical validation—and of accounting for population density—to accurately evaluate risk and enhance recovery programs for at-risk populations. As threats to endangered species continue to mount, biologists will need ever more robust methods to estimate extinction risk. Unfortunately, field biologists typically can't generate the large, high-quality datasets that led to the precise predictions reported here. Conservation efforts will depend on developing methods of generating reliable predictions with the limited data available from the field.

